# Das Münchner Streetwork-Projekt „Senior*innen aufsuchen im Viertel durch Expert*innen“ (SAVE) – eine multimethodische Evaluationsstudie

**DOI:** 10.1007/s00391-025-02477-7

**Published:** 2025-08-04

**Authors:** Hanna Klingshirn, Laura Wehr

**Affiliations:** Kompetenzzentrum „Zukunft Alter“, Katholische Stiftungshochschule München, Preysingstr. 95, 81667 München, Deutschland

**Keywords:** Aufsuchende Soziale Arbeit, Niederschwellige Angebote, Ältere Menschen, Stadtteil, Sozialraumorientierung, Outreach social work, Low-threshold services, Older people, Community, Social space orientation

## Abstract

**Hintergrund:**

In München bieten Alten- und Service-Zentren (ASZ) präventive und versorgende Angebote für ältere Menschen an. Da die Komm-Struktur bestehender Hilfsangebote eine Barriere darstellt, wurde das Streetwork-Projekt „Senior*innen aufsuchen im Viertel durch Expert*innen“ (SAVE) ins Leben gerufen. Seit 2019 beraten SAVE-Fachkräfte ältere Menschen, die sich im öffentlichen Raum aufhalten und einen Unterstützungsbedarf vermuten lassen. Ziel unserer Studie war es, die Umsetzung von SAVE zu untersuchen, um daraus Handlungsempfehlungen abzuleiten.

**Methode:**

Die SAVE-Evaluation basiert auf einem mehrstufigen, multimethodischen Erhebungsverfahren mit partizipativem Ansatz. Zunächst wurden Sekundärdaten (SAVE-Statistik, Projektdokumente) analytisch aufgearbeitet. Des Weiteren fanden teilnehmende Beobachtungen (*n* = 4), eine Fokusgruppe (*n* = 6) sowie Interviews mit SAVE-Fachkräften (*n* = 4) und ASZ-Leitungen (*n* = 5) statt. Zur Integration der Daten wurden die jeweiligen Ergebnisse gegenübergestellt, trianguliert und gemeinsam interpretiert.

**Ergebnisse:**

Das Projekt erreichte laut Statistik (Zeitraum: Januar 2020 bis Juni 2023) 1546 Personen (57 % Frauen; 2847 Kontakte). Häufige Problemlagen waren körperliche und psychische Erkrankungen, finanzielle Probleme und Einsamkeit. Die abgeleiteten Handlungsempfehlungen adressieren folgende Themen: SAVE-Anforderungsprofil, Basisausstattung, Implementierung, Orientierungsphase, Außenauftritt und kollegiale Zusammenarbeit.

**Diskussion:**

Das Projekt SAVE bietet eine innovative Möglichkeit, ältere Menschen in ihrem Stadtteil zu erreichen, indem Fachkräfte Informationen bereitstellen, Beziehungen aufbauen, vor Ort beraten und weitere Hilfen vermitteln. Das Projekt kann als wichtige Maßnahme zur Prävention von Notlagen älterer Menschen betrachtet werden.

**Zusatzmaterial online:**

Zusätzliche Informationen sind in der Online-Version dieses Artikels (10.1007/s00391-025-02477-7) enthalten.

## Abstract (en)

**Background:**

Old age and service centers (ASZ) in Munich offer preventive and care services for older people. As initiating contact with existing support services is a barrier, the street work project “Senior citizens visited by experts in the community” (SAVE) was introduced. Since 2019 SAVE experts have been providing advice to older people who are in public spaces and appear to be in need of support. The aim of our study was to examine the implementation of SAVE to identify recommendations for action.

**Method:**

The SAVE evaluation is based on a mixed methods multistage design with a participatory approach. Firstly, secondary data (SAVE statistics, project documents) were analyzed. In addition, participant observations (*n* = 4), a focus group (*n* = 6) and interviews with SAVE experts (*n* = 4) and ASZ managers (*n* = 5) were conducted. To integrate the data the respective results were compared, triangulated and jointly interpreted.

**Results:**

According to the statistics (period: January 2020–June 2023) the project reached 1546 people (57% women, 2847 contacts). Common problems identified included physical and mental illnesses, financial difficulties and loneliness. The derived recommendations for action address the following topics: SAVE requirements profile, basic equipment, implementation, orientation phase, public awareness and collegial cooperation.

**Conclusion:**

The SAVE project offers an innovative way of reaching older people in their community by providing information, building relationships, giving on-site counselling and providing additional support. The project can be seen as an important measure to prevent distress among older people.

## Hintergrund

Der demografische Wandel führt dazu, dass das Thema „Älterwerden“ gesellschaftlich an Bedeutung gewinnt. Bei vielen Menschen verändern sich im Alter das körperliche und psychische Wohlbefinden [1]. Zudem führen Einsamkeit, soziale Isolation, Pflegebedarfe oder finanzielle Schwierigkeiten oftmals zu Belastungen [2, 3]. Viele ältere Menschen benötigen Unterstützung in ihrer unmittelbaren Lebenswelt, weshalb sozialraumorientierte Zugänge der Sozialen Arbeit mittels aufsuchender Angebote an Bedeutung gewinnen [4, 5].

Streetwork hat sich im deutschsprachigen Raum seit den späten 1970er-Jahren als fester Bestandteil der Sozialen Arbeit etabliert. Sie richtet sich gezielt an problematisierte oder gesellschaftlich ausgegrenzte Gruppen im öffentlichen Raum. Der Ansatz zeichnet sich durch seine bedingungslose Zugänglichkeit aus und ermöglicht Betroffenen den Zugang zu sozialen Hilfsangeboten und staatlichen Ressourcen. Zentrales Element ist der Aufbau von Vertrauen und tragfähigen Beziehungen – als Grundlage für weiterführende Unterstützungsangebote. Traditionell sind eher jüngere Menschen die Zielgruppe der Streetwork, obwohl in den letzten Jahren das Interesse wächst, auch ältere Menschen stärker in den Blick zu nehmen – insbesondere solche, die vereinsamt, gesundheitlich eingeschränkt oder von Armut betroffen sind [5].

Bei der Gestaltung quartiersbezogener Angebote setzt die Landeshauptstadt München auf ihre stadtweiten Alten- und Service-Zentren (ASZ) sowie auf das dort angebundene Streetwork-Projekt „Senior*innen aufsuchen im Viertel durch Expert*innen“ (SAVE) [6, 7]. Seit Eröffnung des ersten Münchner ASZ im Jahr 1979 ermöglichen die dezentral organisierten Einrichtungen präventive und versorgende Leistungen für ältere Menschen und ihre Angehörigen in ihrem Stadtteil [6]. Die Angebote der ASZ variieren je nach Einrichtung, bieten jedoch stets die Möglichkeit, Kontakte zu knüpfen, an Kursen oder Veranstaltungen teilzunehmen, sowie Beratung und Unterstützung in verschiedenen Aspekten des Älterwerdens in Anspruch zu nehmen [6].

**Abb. 1:**
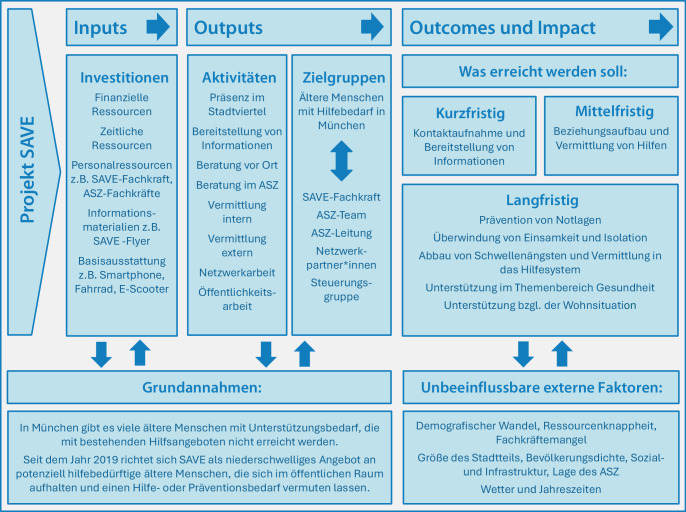
Das SAVE-Konzept im Überblick

Doch nicht alle älteren Menschen, die Unterstützung benötigen, finden den Weg in ein ASZ oder zu anderen Einrichtungen: Oft sind sie nicht über die vorhandenen Angebote informiert, von der bestehenden Komm-Struktur überfordert oder meiden entsprechende Orte aus Scham oder Misstrauen. Besonders betroffen sind jene, die mit mehreren Herausforderungen wie Armut, eingeschränkter Mobilität oder kognitiven Beeinträchtigungen kämpfen. Für all diese Menschen wurde im Jahr 2019 das niedrigschwellige Projekt SAVE (Abb. 1) ins Leben gerufen [7].

Im Jahr 2022 war das Projekt bereits in 9 ASZ implementiert. SAVE richtet sich an ältere Menschen, die sich im öffentlichen Raum aufhalten und einen Unterstützungsbedarf vermuten lassen. Die SAVE-Fachkräfte sind in ihrem jeweiligen Stadtteil unterwegs, um Beziehungen zu diesen Personen aufzubauen und sie auf bestehende Hilfsangebote aufmerksam zu machen. Sie gehen aktiv auf ältere Menschen zu, beraten bei Bedarf vor Ort oder vermitteln an das zuständige ASZ bzw. an andere Fachdienste [7].

Das Ziel unserer SAVE-Evaluationsstudie war es, die Umsetzung des Projekts zu untersuchen, um daraus Handlungsempfehlungen für die zukünftige Durchführung von SAVE in München abzuleiten. Der vollständige Evaluationsbericht wurde zusammen mit der Beschlussvorlage „Ausbau der offenen Altenhilfe“ veröffentlicht [8].

## Methodik

### Studiendesign

**Abb. 2:**
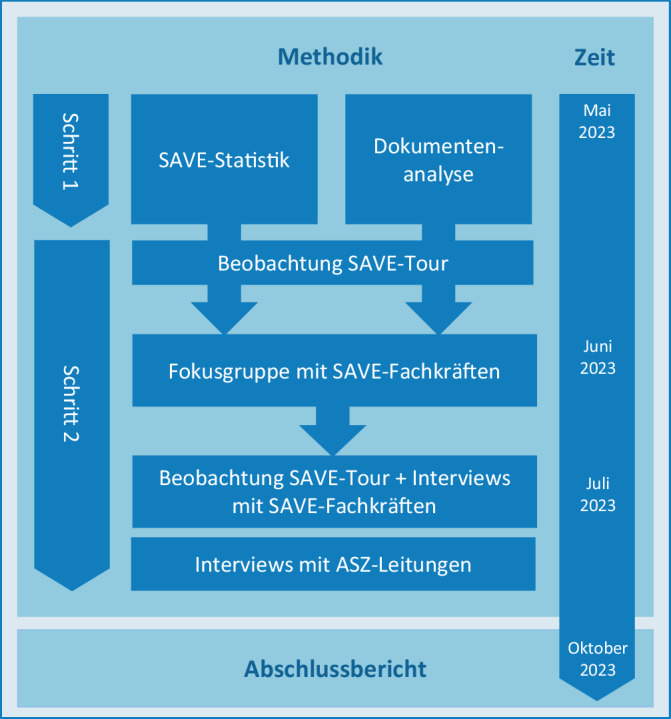
Spezifische Ziele und Methoden der SAVE-Evaluation

Die SAVE-Evaluation basiert auf einem mehrstufigen, multimethodischen und partizipativen Forschungsansatz, der das erfahrungsbasierte Praxiswissen der Fachkräfte in den Mittelpunkt stellt: Im forschenden Dialog mit den am SAVE-Projekt beteiligten Personen – insbesondere den SAVE-Fachkräften und ASZ-Leitungen – konnten die strukturellen Herausforderungen des Streetwork-Projekts rekapituliert und praxisnahe Handlungsempfehlungen für die zukünftige Durchführung von SAVE entwickelt werden – was wiederum die Handlungskompetenz der Beteiligten perspektivisch stärkte [9]. Durch die Anwendung einer mehrstufigen, multimethodischen Vorgehensweise konnten zudem spezifische Teilaspekte der Evaluation vertieft und vorläufige Ergebnisse fundiert abgesichert werden (Abb. 2).

### Datenerhebung

Im ersten Schritt der Datenerhebung wurden vorhandene Daten und Dokumente (Sekundärdaten), wie die SAVE-Statistik (deskriptive statistische Analyse), das SAVE-Konzept, Protokolle und Stadtratsbeschlüsse (qualitative Inhaltsanalyse der Dokumente), analytisch aufgearbeitet.

Der zweite Schritt, auf den wir uns in diesem Artikel fokussieren, gründete auf einer Fokusgruppe mit den SAVE-Fachkräften (*n* = 6), bei der erste Empfehlungen für die Zukunft von SAVE erarbeitet wurden. Des Weiteren wurden qualitative Interviews mit SAVE-Fachkräften (*n* = 4), teilnehmende Beobachtungen auf SAVE-Touren (*n* = 4) sowie Interviews mit ausgewählten ASZ-Leitungen (*n* = 5, eine Person je Trägerschaft) durchgeführt. Vor der Durchführung der Erhebung wurden alle Teilnehmenden über Ziel und Ablauf der Studie informiert, und ein schriftliches Einverständnis zur Studienteilnahme wurde eingeholt. Eine tabellarische Darstellung der Erhebungsinstrumente findet sich im Zusatzmaterial online: Appendix 1.

#### Durchführung der Fokusgruppe

Die 3‑stündige Fokusgruppendiskussion, an der 6 SAVE-Fachkräfte teilnahmen, wurde in Form eines Workshops gestaltet [10]. Dies ermöglichte es den SAVE-Fachkräften, ihre Erfahrungen, Wahrnehmungen und Ideen zum SAVE-Projekt miteinander zu diskutieren, um daraus Handlungsempfehlungen für die Weiterentwicklung von SAVE abzuleiten (gleichberechtigte Zusammenarbeit und gemeinsame Entscheidungsfindung).

Die zentralen Ergebnisse der Fokusgruppe wurden schriftlich und fotografisch in einem Protokoll festgehalten, das die Teilnehmenden im Nachgang prüfen und kommentieren konnten (Reflexivität und Transparenz).

#### Durchführung der qualitativen Interviews

Vier SAVE-Fachkräfte, die terminbedingt nicht an der Fokusgruppe teilnehmen konnten, wurden mittels Leitfadeninterviews befragt. Um die Sichtweisen der beteiligten ASZ-Leitungen auf das SAVE-Projekt abzubilden, wurden außerdem Leitfadeninterviews mit 5 ASZ-Leitungen geführt [11]. Der Gesprächsleitfaden fokussierte auf die konkreten Erfahrungen der SAVE-Fachkräfte in ihrem Arbeitsalltag; zugleich wurde aber auch den thematischen Schwerpunktsetzungen der Teilnehmenden gefolgt.

Die Interviews wurden von 2 erfahrenen wissenschaftlichen Mitarbeiterinnen (H.K., L.W.) durchgeführt und mit Zustimmung der Teilnehmenden digital aufgezeichnet. Durchschnittlich dauerten die Interviews mit den SAVE-Fachkräften 64 (min. 52; max. 80; Median 62) min, die mit den ASZ-Leitungen 72 (min. 60; max. 83; Median 68) min.

### Datenanalyse

Die qualitative Inhaltsanalyse der wörtlich transkribierten Interviews erfolgte mithilfe des Analysetools MAXQDA (VERBI Software GmbH, Berlin, Deutschland) [12]. Induktiv wurden zentrale Themen im Datenmaterial identifiziert, analysiert und kritisch diskutiert. Im Analyseprozess wurde ein Kategoriensystem mit Ober- und Unterkategorien entwickelt; generalisierte Aussagen wurden zu einzelnen Kategorien zusammengefasst [13]. Quantitative Daten (z. B. Alter, Geschlecht) wurden deskriptiv ausgewertet.

Zur Integration der Daten wurden die Ergebnisse aus den unterschiedlichen Auswertungsschritten zusammengetragen und trianguliert. Um den unterschiedlichen Perspektiven und Interpretationsmöglichkeiten Rechnung zu tragen, wurde ein dialogisch-diskutierendes Verfahren im Zweierteam (L.W., H.K.) angewandt, wodurch die Stichhaltigkeit der Analyse zusätzlich abgesichert werden konnte.

## Ergebnisse

### Beschreibung der Befragten

Die 10 SAVE-Fachkräfte (Frauen: *n* = 7; Männer: *n* = 3) hatten ein durchschnittliches Alter von 44 (min. 23; max. 62; Median 49) Jahren und durchschnittlich 12 (min. 1; max. 37; Median 5) Jahre Berufserfahrung in der Sozialen Arbeit. Die ASZ-Leitungen (Frauen: *n* = 3; Männer; *n* = 2) hatten ein durchschnittliches Alter von 48 (min. 33; max. 64; Median 48) Jahren und durchschnittlich 22 (min. 9; max. 37; Median 21) Jahre Berufserfahrung in der Sozialen Arbeit.

### Wer bisher mit SAVE erreicht wurde

Mit dem Projekt SAVE wurden 1546 Personen erreicht (Statistik von 01.01.2020 bis 30.06.2023). Insgesamt wurden 2847 Kontakte dokumentiert, wobei die Anzahl der Kontakte pro Person zwischen einem und 153 Kontakten (Durchschnitt: 1,8; Median: 1) variiert.

**Abb. 3:**
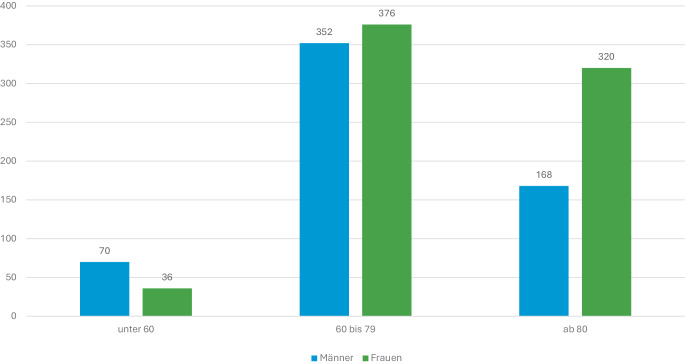
Erreichte Personen, differenziert nach Altersgruppen und Geschlecht

Altersangaben wurden bei insgesamt 1322 Personen dokumentiert (Abb. 3). Demnach bilden die größte Altersgruppe die 60- bis 79-Jährigen mit insgesamt 728 Personen (Frauen 52 %). In der Altersgruppe der über 80-Jährigen sind mit 66 % deutlich mehr Frauen vertreten, während es bei den unter 60-Jährigen mit 34 % deutlich weniger Frauen sind.

### Integration der Ergebnisse

Im Folgenden werden die Ergebnisse der verschiedenen Teilstudien auf der Ebene der Oberkategorien dargestellt. Die jeweiligen Unterkategorien werden im Zusatzmaterial online: Appendix 2 detailliert beschrieben und mit Beispielzitaten belegt.

#### Auf der Straße: als SAVE-Fachkraft unterwegs im Stadtviertel

Bereits bei den teilnehmenden Beobachtungen während der SAVE-Touren wurde ersichtlich, dass die SAVE-Fachkräfte grundsätzlich offen für alle Begegnungen mit älteren Menschen sind und unabhängig von äußeren Merkmalen, wie z. B. Geschlecht oder Hautfarbe, auf alle älteren Menschen zugehen. Diese Haltung stellt ein zentrales Prinzip ihrer Streetwork-Tätigkeit dar. Wie auf der Straße pragmatisch mit etwaigen Sprachbarrieren umgegangen wird, beschreibt eine SAVE-Fachkraft:

„[Das mit den] Sprachkenntnissen, das geht dann schon. […] Wenn ich merke, okay, der spricht jetzt, keine Ahnung, Russisch, dann kann ich sagen, da hab’ ich auch was dabei. Geht auch. Aber dann versucht man es halt im Kontakt mit Händen und Füßen und [hat] dann die Informationsmaterialien. […] Wir haben auch Kontakt zur Migrationsberatung, wir haben Flyer in verschiedenen Sprachen, was möglich wäre. Und dadurch, dass ich ein Diensthandy habe, kann man theoretisch auch einfach mit Google Übersetzer [arbeiten], […] wenn es notwendig ist.“ (Int2-ASZ‑F, Pos. 117-121)

Durch die direkte Ansprache im öffentlichen Raum (z. B. Straßen, Plätze oder Parks) werden auch Menschen erreicht, die wenig Vertrauen in institutionelle Unterstützungsangebote haben. Eine ASZ-Leitung beschreibt den Gedanken von SAVE wie folgt:

„Die Idee […] von SAVE [ist], dass man […] Leute anspricht, auf die Gefahr hin, dass die dann sagen, ‚Was wollen Sie von mir?‘ oder ‚Sehe ich wirklich so alt aus?‘ oder ‚Was wollen Sie mir verkaufen?‘ oder ‚Lasst mich einfach in Ruhe!‘. So reagieren die [Leute] ja öfter. Aber viel öfter reagieren sie positiv überrascht, dass sich jemand für sie interessiert und jemand fragt, wie es ihnen geht. Das hat sie schon lang keiner mehr gefragt.“ (Int5-ASZ‑L, Pos. 30)

Um verlässliche Beziehungen aufbauen zu können, bewegen sich die SAVE-Fachkräfte auf festgelegten Routen durch das Stadtviertel. Allerdings zeigt die Praxis, dass wechselnde Jahreszeiten und Wetterlagen eine gewisse Flexibilität im Arbeitsalltag erfordern. Beim Erstkontakt verfolgen alle SAVE-Fachkräfte ein ähnliches Vorgehen: Sie gehen aktiv auf ältere Menschen zu, stellen sich vor und weisen, oftmals in Verbindung mit dem Programmheft mit aktuellen Angeboten, auf ihre Zugehörigkeit zum ASZ hin.

Gerade der Erstkontakt erfordert Kreativität, Offenheit, Flexibilität und eine hohe Frustrationstoleranz: Denn die Fachkräfte müssen sich immer wieder bewusst machen, dass nicht jede angesprochene Person einen Hilfebedarf hat oder sofort äußert. Durch die stete Präsenz im Viertel und Wiedererkennungseffekte gelingt es jedoch oft, Vertrauen zu schaffen und eine Beziehung aufzubauen. Als Fachkraft professionell und damit „sichtbar“ aufzutreten und gleichzeitig die Anonymität der älteren Menschen im Sozialraum zu wahren (Unsichtbarkeit), wird als besondere Herausforderung erlebt. Seriosität und Transparenz stellen die SAVE-Fachkräfte durch Materialien wie den SAVE-Flyer mit Foto und Kontaktdaten her.

Nach dem Kennenlernen auf der Straße erfolgt bei Bedarf eine weiterführende Beratung. Problemlagen wie körperliche und psychische Erkrankungen, finanzielle Schwierigkeiten oder Einsamkeit wurden häufig dokumentiert, obgleich im Mittelpunkt einer Beratung immer die individuelle Situation der hilfesuchenden Person steht. Daraus ergibt sich der Anspruch an die SAVE-Fachkräfte, in unterschiedlichsten Themenbereichen (z. B. Einsamkeit, Pflege, medizinische Versorgung, Wohnen, hauswirtschaftliche Versorgung, finanzielle Hilfen) kompetent Auskunft zu geben, um in das lokale Hilfesystem vermitteln zu können.

#### Jenseits der Straße: SAVE-Arbeit im ASZ

Deutlich wurde zudem, dass das SAVE-Konzept eng mit den ASZ-Strukturen verzahnt werden muss, damit die erfolgreiche Umsetzung gelingt. Förderliche institutionelle Rahmenbedingungen sind dabei entscheidend, denn SAVE ist kein Ein-Personen-Projekt: Es braucht die Unterstützung von ASZ-Leitung und -Team.

Im Zuge der Vorbereitung ihrer Tour steht jede SAVE-Fachkraft vor der Aufgabe, den eigenen Stadtteil systematisch zu erfassen. Stadtteilbegehungen sowie Infrastrukturdatenanalysen haben sich als nützlich erwiesen, um ein umfassendes Verständnis der lokalen Versorgungsstruktur zu gewinnen und zielführende Routen zu entwickeln. Bei den Begehungen können zudem potenzielle Netzwerkpartner*innen identifiziert, Kooperationen angestoßen und das Projekt im öffentlichen Raum sichtbar gemacht werden. Eine ASZ-Leitung berichtet dazu:

„Durch die Arbeit für SAVE hat sie [unsere SAVE-Fachkraft] […] unglaublich viel Öffentlichkeitsarbeit gemacht und sich vorgestellt und war bei XY. Und dadurch sind dann Kontakte entstanden, die tatsächlich bis jetzt halten. […] Und das ist wiederum fürs ASZ gut. […] Also, da hat sie nochmal ein Netzwerk aufgebaut, was wir hier so definitiv nicht hingekriegt hätten, nur in unserer Arbeitszeit. Das ist ein Riesenvorteil geworden, das Haus durch ihre Arbeit einfach präsent zu machen.“ (Int1-ASZ‑L, Pos. 39-41)

Zudem ist ein zentraler Bestandteil der SAVE-Arbeit, Informationen bereitzustellen und Hilfen zu vermitteln. Als „Türöffner“ sind die SAVE-Fachkräfte dafür zuständig, Hemmschwellen abzubauen und den Weg ins Hilfesystem zu ebnen.

#### Für die Straße: Standortbestimmung und Zukunftsperspektiven

**Abb. 4:**
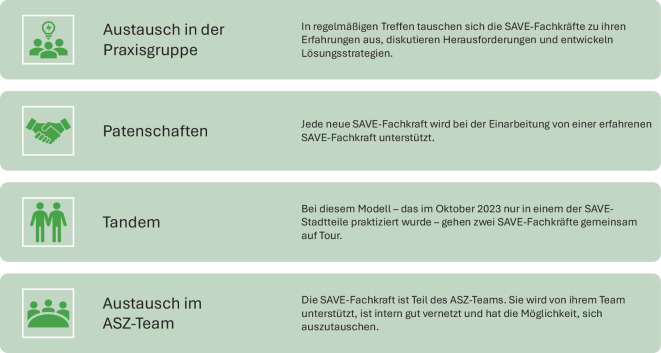
Strategien zur Förderung von Teamarbeit und kollegialem Austausch

Wie die SAVE-Fachkräfte berichteten, sahen sie sich besonders zu Beginn ihrer Tätigkeit mit der Herausforderung konfrontiert, eine neue fachliche Rolle in einem Pilotprojekt auszufüllen. Einerseits bot sich dadurch die Möglichkeit, innovative Ansätze zu verfolgen; andererseits fehlten etablierte Strukturen zur Implementierung. Vielfach wurde betont, wie wichtig die Unterstützung des Teams und der kollegiale Austausch für die SAVE-Tätigkeit seien und welche Strategien sich dabei bewährt hätten (Abb. 4).

Ein viel diskutiertes Thema ist die Weitervermittlung der SAVE-Klientel bei gleichzeitig begrenzten Ressourcen. Viele ASZ-Kurse und -Angebote sind vollbesetzt, der soziale Mittagstisch oft ausgebucht und die Nachfrage nach Beratungsterminen hoch. Insofern muss perspektivisch sichergestellt werden, dass zusätzliche Ressourcen für die Weitervermittlung der SAVE-Klientel zur Verfügung stehen.

**Abb. 5:**
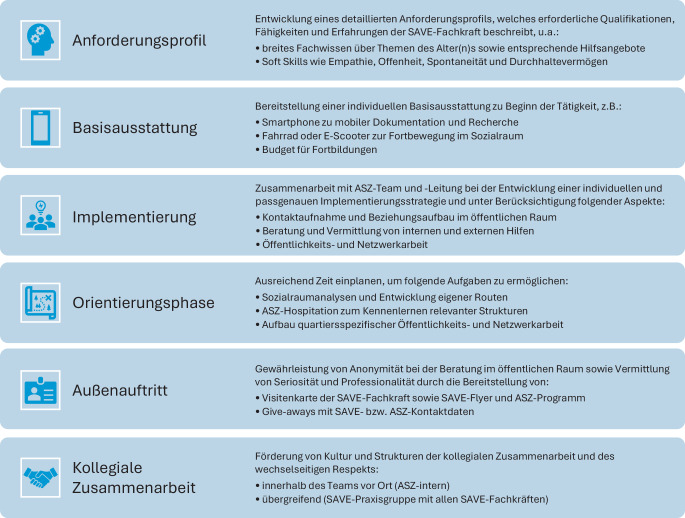
Handlungsempfehlungen für die Zukunft von SAVE

Münchens Stadtteile sind sozialräumlich sehr unterschiedlich strukturiert. Wie die Befragten berichteten, hängt der Erfolg von SAVE maßgeblich von der Anpassung des Angebots an die spezifischen Bedarfe der einzelnen Stadtteile ab. Besonders die ASZ in flächenmäßig großen Stadtbezirken stehen vor der schwierigen Aufgabe, verschiedene Quartiere innerhalb eines Einzugsgebiets versorgen zu müssen. Zukünftig sollte deshalb berücksichtigt werden, dass diese strukturelle Herausforderung auch eine spezielle Ausstattung (z. B. Fahrrad oder E‑Scooter) erfordert. Die Evaluation ergab wichtige Handlungsempfehlungen für die zukünftige Durchführung von SAVE in München (Abb. 5**)**.

## Diskussion

Die beschriebene Studie evaluierte erstmals SAVE, ein einzigartiges Streetwork-Projekt für ältere Menschen in München. Mit dem Projekt konnten – trotz erschwerter Bedingungen aufgrund der Coronapandemie – 1546 ältere Menschen erreicht werden (Zeitraum: Januar 2020 bis Juni 2023). Deutlich wurde zudem, mit welcher Vielzahl an Bedarfen die SAVE-Fachkräfte konfrontiert sind. Auf ihren Touren begegnen sie verschiedensten Problemlagen, dazu zählen Einsamkeit, soziale Isolation, körperliche und psychische Erkrankungen sowie finanzielle Sorgen. Gerade in Großstädten wie München stellt Altersarmut, verbunden mit der Nichtinanspruchnahme von Grundsicherungsleistungen, ein zunehmendes Problem dar [3, 14]. Die vorliegende Evaluation zeigt, dass mit dem SAVE-Projekt neben Menschen in Armutslagen auch Menschen mit Migrationsgeschichte erreicht werden können. Durch den proaktiven Charakter des Angebots werden zudem viele Männer angesprochen, die ansonsten kaum für Angebote der Prävention und Gesundheitsförderung zu gewinnen sind [15]. Im Sinne des 9. Altersberichts geht das SAVE-Projekt insbesondere auf die vielfältigen Lebenslagen und ungleichen Teilhabechancen älterer Menschen ein [16].

Wer als SAVE-Fachkraft im Stadtviertel unterwegs ist, benötigt neben breitem Wissen über die Angebote des lokalen Hilfesystems auch spezifische Soft Skills wie Empathie und Spontaneität . Aus anderen Bereichen der Streetwork ist bekannt, dass eine offene Grundhaltung bei der Kontaktaufnahme im öffentlichen Raum ein zentrales Erfolgskriterium darstellt [17]. Weiterhin gilt das „Aufsuchen“ im Tandem als ein Qualitätsmerkmal [18], das sich auch im SAVE-Projekt bewährt hat. Grundsätzlich stellt die Anonymität eine zentrale Handlungsmaxime der Streetwork dar, die durch das Herstellen „flüchtiger Beratungsräume“ im öffentlichen Raum allerdings nur teilweise gewährleistet werden kann [19]. Im Rahmen von SAVE besteht die Möglichkeit, die Beratung von der Straße in den geschützten Raum des ASZ zu verlagern.

Die Ergebnisse der SAVE-Evaluation verweisen zudem auf die große Bedeutung von vorbereitenden Sozialraumanalysen und damit verbundener Netzwerk- und Öffentlichkeitsarbeit [20]. Als zentrale Faktoren für eine erfolgreiche Implementierung wurden die konzeptionelle Verzahnung von SAVE mit dem assoziierten ASZ sowie die konstruktive Zusammenarbeit im Team identifiziert. In Bezug auf die Zukunft von SAVE sind sich alle Befragten einig: Das Projekt ist eine wichtige Maßnahme für die Prävention von Notlagen älterer Menschen in München und sollte daher unbedingt weiterausgebaut werden.

### Limitationen

Aus Gründen der Vertraulichkeit wurden die älteren Menschen, die mit SAVE in Verbindung stehen, nicht persönlich befragt, sondern als Personengruppe lediglich über die SAVE-Statistik rekonstruiert. Bei der Interpretation der Statistik ist zudem zu beachten, dass einige Angaben von den SAVE-Fachkräften geschätzt wurden (z. B. Alter), was Verzerrungen ergeben könnte.

Zudem lässt sich auch in dieser multimethodischen Studie das Phänomen sozial erwünschter Antworten nicht vollständig ausschließen – obwohl sich die Teilnehmenden offen, freimütig und kritisch äußerten.

### Schlussfolgerungen

Viele ältere Menschen, die in Großstädten leben, kämpfen mit finanziellen Sorgen, Einsamkeit und gesundheitlichen Einschränkungen, finden aber nicht den Weg in das städtische Hilfesystem. Das Projekt SAVE zeigt, wie innovative Ansätze in der aufsuchenden Sozialen Arbeit erfolgreich umgesetzt werden können. Um Vertrauen aufzubauen und Hilfsangebote zu vermitteln, sind individuelle und kreative Lösungsstrategien ebenso unerlässlich wie Einfühlungsvermögen und Geduld. Als niederschwelliges Streetwork-Angebot für ältere Menschen kann SAVE ein Vorbild für ähnliche Projekte sein.

## Fazit für die Praxis

- Das Projekt SAVE ist ein niederschwelliges Streetwork-Angebot für ansonsten schwer erreichbare ältere Menschen im städtischen Raum.
- Bei der Implementierung von SAVE vor Ort muss eine Anpassung an die Besonderheiten des jeweiligen Stadtteils erfolgen. Sozialraumanalysen in Kombination mit Netzwerk- und Öffentlichkeitsarbeit sind dabei zentrale Werkzeuge.
- Die Unterstützung der SAVE-Fachkraft durch das ASZ-Team und deren Leitung ist entscheidend für die erfolgreiche Umsetzung von SAVE.

## Supplementary Information

Appendix 1: Überblick zu den qualitativen Erhebungsmethoden und Inhalt der Leitfäden

Appendix 2: Beschreibung der Unterkategorien

## Data Availability

Die Daten können aus Datenschutzgründen nicht zur Verfügung gestellt werden.
