# Supplementary material for: The Munich street work project “Senior citizens visited by experts in the community” (SAVE)—A mixed methods evaluation study
Source: Z Gerontol Geriatr. 2025 Aug 4;59(2):140–7. [Article in German] doi: 10.1007/s00391-025-02477-7 (PMC12953350; doi:10.1007/s00391-025-02477-7)
Supplement: Supplementary file 1 — Appendix 1: Überblick zu den qualitativen Erhebungsmethoden und Inhalt der Leitfäden [file 391_2025_2477_MOESM1_ESM.pdf]

Appendix 1 Überblick zu den qualitativen Erhebungsmethoden und Inhalt der Leitfäden

Erhebungsmethoden	Themenbereiche	Kernfragen/Gesprächsimpulse
**Fokusgruppe mit SAVE-Fachkräften**	Gesprächsimpulse zum Einstieg	Steckbrief: Informationen zu Person und Stadtteil<br>Arbeitsblatt: „Bitte ergänzen Sie: SAVE bedeutet für mich…“<br>Stimmungsbild: In welchen SAVE-Zielbereichen sind Sie am häufigsten tätig?
	Arbeitsalltag	Welche Erfahrungen haben Sie im Arbeitsalltag als SAVE-Fachkraft gemacht? Was hat sich bewährt? Was war schwierig? Was brauchen Sie für die Zukunft?
	Statistik und Dokumentation	Welche Angaben benötigt es aus Ihrer Sicht für eine gute SAVE-Dokumentation? Bitte geben Sie drei Empfehlungen für die Weiterentwicklung der SAVE-Statistik.
	Tätigkeitsbeginn	Was braucht es aus Ihrer Sicht für einen guten Start in die Arbeit als SAVE-Fachkraft?
	Handlungsempfehlungen	Zusammenfassung der bisherigen Diskussionspunkte; gemeinsame Formulierung von Handlungsempfehlungen
	Offene Frage zum Abschluss	Wenn ich mir für meine SAVE-Arbeit etwas wünschen könnte, dann wäre das…
**Interviews mit SAVE-Fachkräften**	Gesprächsimpuls zum Einstieg	Erzählen Sie doch bitte, seit wann Sie in Ihrem ASZ tätig sind und welche Tätigkeiten im ASZ Sie neben Ihrer Arbeit als SAVE-Fachkraft ausführen.
	Arbeitsalltag	Bitte beschreiben Sie möglichst konkret, wie ein typischer SAVE-Tag bei Ihnen aussieht.
	Tätigkeitsbeginn	Denken Sie bitte an den Beginn Ihrer Tätigkeit als SAVE-Fachkraft zurück. Was braucht es für einen guten Start in die Arbeit als SAVE-Fachkraft?
	Handlungsempfehlungen	Bitte fassen Sie noch einmal zusammen, welche wesentlichen Empfehlungen Sie für den weiteren Ausbau von SAVE geben möchten.
	Offene Frage zum Abschluss	Gibt es noch etwas, das Sie uns mitteilen möchten?
**Interviews mit ASZ-Leitungen**	Gesprächsimpuls zum Einstieg	Erzählen Sie doch bitte, seit wann Sie in Ihrem ASZ als Leitung tätig sind und welche Aufgaben mit Ihrer Position verbunden sind.
	Erste Erfahrungen mit SAVE	Nun würde ich gerne erfahren, wie Sie die Einführung von SAVE erlebt haben.
	Zusammenarbeit mit der SAVE-Fachkraft	Bitte beschreiben Sie möglichst konkret, wie Sie als ASZ-Leitung mit der SAVE-Fachkraft zusammenarbeiten.
	Verzahnung SAVE-ASZ-Arbeit	Nun würde ich gerne wissen, wie Sie die Auswirkungen von SAVE auf die allgemeine ASZ-Arbeit erleben.
	Handlungsempfehlungen	Bitte fassen Sie noch einmal zusammen, welche wesentlichen Empfehlungen Sie für den weiteren Ausbau von SAVE geben möchten.
	Offene Frage zum Abschluss	Gibt es noch etwas, das Sie uns mitteilen möchten?
