# Supplementary material for: The Munich street work project “Senior citizens visited by experts in the community” (SAVE)—A mixed methods evaluation study
Source: Z Gerontol Geriatr. 2025 Aug 4;59(2):140–7. [Article in German] doi: 10.1007/s00391-025-02477-7 (PMC12953350; doi:10.1007/s00391-025-02477-7)
Supplement: Supplementary file 2 — Appendix 2: Beschreibung der Unterkategorien [file 391_2025_2477_MOESM2_ESM.pdf]

DAS MÜNCHNER STREETWORK-PROJEKT „SENIOR*INNEN AUFSUCHEN IM VIERTEL DURCH EXPERT*INNEN“ (SAVE) – EINE MULTIMETHODISCHE EVALUATIONSSTUDIE

Appendix 2: Beschreibung der Unterkategorien

Dr. Hanna Klingshirn und Dr. Laura Wehr

KOMPETENZZENTRUM »ZUKUNFT ALTER«
KATHOLISCHE STIFTUNGSHOCHSCHULE MÜNCHEN

Auf der Straße: als SAVE-Fachkraft unterwegs im Stadtviertel

Wer braucht SAVE? Zur Frage der „richtigen“ Zielgruppe

Die SAVE-Fachkräfte begegnen älteren Menschen auf ihren Touren mit einer grundlegend neutralen Haltung. Auch wenn sie auf ihren Touren gezielt Ausschau nach Anzeichen von Hilfebedarf und sozialen Notlagen halten, sind sie grundsätzlich offen für alle Begegnungen. Durch die direkte Ansprache im öffentlichen Raum werden nicht nur Menschen in prekären Lebenssituationen erreicht, sondern auch jene, die kein Vertrauen in öffentliche Hilfsangebote haben und diese entsprechend meiden.

Beispielzitat

„Die Idee [...] von SAVE [ist], dass man [...] Leute anspricht, auf die Gefahr hin, dass die dann sagen, ‚Was wollen Sie von mir?‘, oder ‚Sehe ich wirklich so alt aus?‘, oder ‚Was wollen Sie mir verkaufen?‘, oder ‚Lasst mich einfach in Ruhe!‘. So reagieren die [Leute] ja öfter. Aber viel öfter reagieren sie positiv überrascht, dass sich jemand für sie interessiert und jemand fragt, wie es ihnen geht. Das hat sie schon lang keiner mehr gefragt.“ (Int5-ASZ-L, Pos. 30)

Von Routen, Orten und Wetterlagen: Wo Begegnungen stattfinden

Begegnungen mit potentiell hilfsbedürftigen älteren Menschen finden im Rahmen von SAVE überwiegend im öffentlichen Raum statt, d.h. auf Straßen, Plätzen oder in Parks des jeweiligen Stadtviertels. Die SAVE-Fachkräfte bewegen sich meist auf festgelegten Routen, an festen Tagen und zu fixen Uhrzeiten durch das Stadtviertel rund um das ASZ. Dies ist sinnvoll, um Verlässlichkeit und Wiedererkennung zu gewährleisten. Allerdings zeigt die Praxis, dass wechselnde Wetterlagen und Jahreszeiten immer wieder akute Anpassungen der Routinen erfordern, weshalb Flexibilität im Vorgehen notwendig ist.

Beispielzitat

„Ich verstehe das System: Feste Uhrzeit/fixe Route, um Kontinuität reinzubringen. Aber de facto ist es nicht umsetzbar. Weil, im Sommer brauch ich nicht um 14.00 Uhr am X-platz sein, weil, da ist es heiß, da ist keiner. Im Winter brauch ich aber auch nicht um neun Uhr morgens am X-platz sein, da ist es zu kalt, da ist auch keiner da. Also, man muss das tatsächlich anpassen.“ (Int2-ASZ-F, Pos. 151)

„Kennen Sie uns eigentlich?“ Herausforderungen beim Erstkontakt

Die SAVE-Fachkräfte verfolgen bei ihren Touren durch das Stadtviertel stets ein ähnliches kommunikatives Vorgehen: Sie begrüßen die Zielpersonen, stellen sich selbst und das SAVE-Projekt vor, verweisen auf das zugehörige ASZ und erkundigen sich nach dem Befinden ihres Gegenübers. Bewährt hat es sich zudem, auf die verschiedenen Angebote des ASZ hinzuweisen. Oftmals stoßen die SAVE-Fachkräfte bereits bei der ersten Kontaktaufnahme auf Interesse, manchmal müssen sie jedoch auch mit Ablehnung umgehen oder Schwellenängste abbauen. Anzuerkennen gilt es auch, dass nicht jede angesprochene Person einen Hilfebedarf hat oder diesen schon bei der ersten Begegnung äußert. Insgesamt erfordert gerade der Erstkontakt große Kreativität, Offenheit, Flexibilität und manchmal auch Frustrationstoleranz.

Beispielzitat

„Am Anfang hab ich immer gesagt: Ich bin vom Alten- […] und Service-Zentrum in der X-straße, kennen Sie uns eigentlich? Und hatte unser Programmheft immer dabei als Lockvogel, als Türöffner. Das Programmheft halte ich da für ganz wichtig. […] Das war durchaus erfolgreich, weil manche Leute, die erst sagten: ‚Hm…‘ – und dann aber doch: ‚Ah, ja, […] ich guck´s mir mal an.‘ Und das ist auch gut, selbst wenn die jetzt nicht am Programm teilnehmen. Weil, da steht ja auch, dass wir Beratung machen, dass wir präventive Hausbesuche machen, dass man uns anrufen kann […]. Da haben sie den Kontakt, den sie eigentlich haben sollten, wenn sie Fragen haben. […] Brauchen tun sie uns vielleicht erst in zwei Jahren. Aber dann fällt´s einem wieder ein: ‚Stimmt, da war doch was.‘“ (Int3-ASZ-F, Pos. 55)

„Einfach Kontakt halten!“ Vertrauens- und Beziehungsarbeit

Nach einem gelungenen Erstkontakt stehen die SAVE-Fachkräfte vor der Aufgabe, eine nachhaltige Beziehung zur Zielperson aufzubauen. Ein erfolgreicher Vertrauens- und Beziehungsaufbau auf der Straße erfordert Aufmerksamkeit, Kontinuität – und entsprechend Zeit und Ausdauer. Um Kontakte zu pflegen und Vertrauen zu schaffen, ist es wichtig, im Viertel präsent zu sein, die älteren Menschen individuell zu begrüßen und Interesse an ihrer Person zu zeigen.

Beispielzitat

„Es ist tatsächlich immer dieses: Einfach den Kontakt halten. Auch wenn man jetzt x Kontakte hat, wo es nur ums ‚Grüß dich, alles in Ordnung, wie geht’s?‘ [geht], aber irgendwann kommt ein Thema. […] Irgendwann kommt dann: ‚Ich hab eine Frage zur Rente.‘ Und dann kann man das schnell beantworten, auch von Balkon zu Straße.“ (Int2-ASZ-F, Pos. 125)

(Un-)Sichtbarkeit im Stadtviertel

Als hohe Kunst der SAVE-Arbeit erleben die Fachkräfte den Anspruch, einerseits offiziell und mit einer gewissen Präsenz aufzutreten (Sichtbarkeit) und anderseits die Anonymität der älteren Menschen im Sozialraum zu wahren (Unsichtbarkeit). Auf der Straße agieren die SAVE-Fachkräfte sensibel und transparent und gehen individuell auf ihr Gegenüber ein. Eine offene Kommunikation sowie der Einsatz von Informationsmaterialien (wie der SAVE-Flyer mit Foto und Kontaktdaten oder das ASZ-Programmheft) unterstützen den seriösen Auftritt und vermitteln dem Gegenüber Sicherheit bei einer gleichzeitigen Wahrung der Anonymität.

Beispielzitat

„Manche Kolleginnen haben am Anfang gemeint, wir müssen wie so eine rote Litfaßsäule durch die Gegend gehen, damit die Leute wissen, das ist SAVE, das ist das ASZ, die kann ich ansprechen. Aber andere haben gemeint, das hat auch viele Nachteile, weil, man muss sich dann immer gegenüber der Umgebung auch outen, weil jeder sieht, oh Gott, jetzt redet die auch schon mit der SAVE-Kraft […], die scheint´s auch nimmer ganz auf die Reihe zu kriegen. [Lacht.] […] Ich glaub nicht, dass man so rot leuchtend durch den Stadtteil gehen sollte, aber schon offen und schnell eine Karte oder ein Namensschild oder einen Flyer zücken. Das find ich schon wichtig, dass man sich nicht so anschleicht von hinten und nach 20 Minuten […] erst damit ankommt. Das fände ich dann übergriffig.“ (Int5-ASZ-L, Pos. 112-114)

Sechs SAVE-Zielbereiche, unzählige Beratungsthemen?

Themen wie körperliche und psychische Erkrankungen, finanzielle Probleme oder Einsamkeit wurden besonders häufig in der Statistik dokumentiert. Im Mittelpunkt von SAVE stehen jedoch stets die individuellen Problemlagen der hilfesuchenden Person. Duch eine passgenaue Beratung auf der Straße ergeben sich zahlreiche Beratungsthemen wodurch die SAVE-Fachkräfte, in vielen Themenbereichen inhaltlich vorbereitet und gesprächsbereit sein müssen. Wesentlich sind dabei auch vertiefte Kenntnisse zu den Angeboten des lokalen Hilfesystems, um als SAVE-Fachkraft adäquat agieren und entsprechende Beratungs- bzw. Unterstützungsangebote vermitteln zu können.

Beispielzitat

„Tatsächlich ist ein großes Thema die Einsamkeit. Das ist ein sehr großes Thema, vor allem für die alleinstehenden Damen und Herren. Dann gibt's noch finanzielle Probleme; hauswirtschaftliche Versorgung ist auch mal ein Problem. Das sind so die Hauptthemen. Und halt auch eingeschränkte Mobilität und welcher Arzt, wer macht Hausbesuche.“ (Int2-ASZ-F, Pos. 41)

Jenseits der Straße: SAVE-Arbeit im ASZ

**Implementieren, adaptieren, verzahnen: SAVE als Bestandteil der ASZ-Arbeit**
Um SAVE erfolgreich durchzuführen, muss das Konzept nahtlos in die Arbeit des ASZ integriert werden. Förderliche institutionelle Rahmenbedingungen sind dabei entscheidend, denn obgleich die SAVE-Fachkraft bei der Implementierung von SAVE eine zentrale Rolle spielt, ist SAVE kein Ein-Personen-Projekt: Für eine erfolgreiche Umsetzung von SAVE braucht es die Unterstützung der ASZ-Leitung und den Rückhalt des gesamten ASZ-Teams.
**Beispielzitat**
„Wenn es die Aufgabe von SAVE ist, die Leute ins Haus zu bringen oder ans Haus anzudocken, dann ist es natürlich wichtig, dass man [als SAVE-Fachkraft] auch weiß, was hier [im ASZ] passiert [...], an wen man sich gegebenenfalls wendet [...].“ (Int3-ASZ-F, Pos. 3)

**Vorbereitungen auf die Tour: Die Sozialraumanalyse**
Jede SAVE-Fachkraft steht vor der Herausforderung, den eigenen Stadtteil als Sozialraum systematisch zu erfassen. Neben amtlichen Statistiken und vorhandenen Wissensbeständen im Team haben sich dafür Stadtteilbegehungen und Sozialraumanalysen als nützlich erwiesen. Diese Methoden helfen den Fachkräften nicht nur dabei, eigene Routen zu entwickeln, sondern auch, den Stadtteil besser kennenzulernen, Netzwerke zu knüpfen und ein umfassendes Verständnis der lokalen Versorgungs- und Infrastruktur zu gewinnen.
**Beispielzitat**
„Der Vorteil war, dass wir zu dem Zeitpunkt, wo ich gestartet bin, [erstmal] eine Stadtteilbegehung gemacht haben. Das heißt, ich bin mit verschiedenen Kollegen vom Team, wir haben den kompletten Stadtteil quasi in Parzellen eingeteilt [...]. Und da bin ich immer mit einer anderen Kollegin mitgegangen, und wir haben uns Straßenzug für Straßenzug den Stadtteil angeschaut. Und das hat mir wahnsinnig viel gebracht, weil, da hast du einfach gesehen, wie die Struktur ist. Wir haben dann aufgeschrieben, was uns so aufgefallen ist: [...] Öffentlicher Nahverkehr, Kommunikationsmöglichkeiten, Gesundheitswesen, Behörden... [...] Dass man einfach schon mal weiß, was gibt’s. Und dann hab ich mir überlegt, wo könnte ich Leute antreffen. Dann hab ich Beobachtungen gemacht zu verschiedenen Uhrzeiten und hab gezählt, wieviel alte Menschen ich treffe, wieviel Leute potenziell Hilfebedarf hätten. Und so hab ich mir [nach und nach] die Routen erarbeitet.“ (Int2-ASZ-F, Pos. 151)

Kooperation im Stadtviertel: Netzwerkarbeit

Um den Prozess der Weitervermittlung von hilfebedürftigen älteren Menschen in das bestehende Hilfesystem zu optimieren, ist eine passgenaue Netzwerkarbeit zentral. Zu den kontinuierlichen Aufgaben der SAVE-Fachkräfte gehört es daher, das SAVE-Projekt nicht nur bei der Zielgruppe der älteren Menschen, sondern auch bei den Akteur*innen im Sozialraum bekannt zu machen. Auf das Stadtviertel abgestimmte Sozialraumanalysen bieten eine erste Möglichkeit, potenzielle Netzwerkpartner*innen zu identifizieren, um daran anschließend gezielt Kontakte zu knüpfen und Kooperationen anzustoßen.

Beispielzitat

„Durch die Arbeit für SAVE hat sie [unsere SAVE-Fachkraft] […] unglaublich viel Öffentlichkeitsarbeit gemacht und sich vorgestellt und war bei XY. Und dadurch sind dann Kontakte entstanden, die tatsächlich bis jetzt halten. […] Und das ist wiederum fürs ASZ gut. […] Also, da hat sie nochmal ein Netzwerk aufgebaut, was wir hier so definitiv nicht hingekriegt hätten, nur in unserer Arbeitszeit. Das ist ein Riesenvorteil geworden, das Haus durch ihre Arbeit einfach präsent zu machen.“ (Int1-ASZ-L, Pos. 39-41)

Sichtbarkeit schaffen: Öffentlichkeitsarbeit

Eng verbunden mit der Netzwerkarbeit ist das Thema Öffentlichkeitsarbeit. Um Sichtbarkeit zu schaffen, wird durch gezielte öffentlichkeitswirksame Maßnahmen und Aktivitäten das Bewusstsein für die Angebote und Möglichkeiten des SAVE-Projekts gestärkt. So kann eine breite Akzeptanz und Unterstützung im Sozialraum erreicht werden.

Beispielzitat

„Ich hab quasi [am Anfang] Öffentlichkeitsarbeit und die SAVE-Arbeit zusammen vernetzt, hab mich bei den einzelnen Einrichtungen vorgestellt, auch mit Termin, und hab die SAVE-Arbeit vorgestellt und die ASZ-Arbeit […].“ (Int2-ASZ-F, Pos. 45)

Türöffner sein: Wege ins Hilfesystem vermitteln

Ein weiterer zentraler Aspekt der SAVE-Arbeit besteht darin, Informationen bereitzustellen und ggf. Hilfen zu vermitteln. Als „Türöffner" sind die SAVE-Fachkräfte dafür verantwortlich, potentielle Unterstützungsbedarfe älterer Menschen zu erkennen und mögliche Hemmschwellen gegenüber Hilfsangeboten abzubauen.

Beispielzitat

„Es gibt Situationen, da berate ich einfach auf der Straße zu den Themen. Wenn ich den großen Ordner dabei hab, hab ich ja alle Informationen schon dabei und kann das quasi vor Ort machen. Es gibt aber auch Situationen, da vermittle ich dann ans ASZ, wir haben eine zentrale Telefonnummer, wenn nicht explizit nach mir gefragt wird, dann übernimmt die Kollegin, die gerade Zeit hat. Mir ist es wichtig, nicht nur ins ASZ zu vermitteln, sondern auch zu anderen Angeboten. Also, ich informiere mich, was bieten die Kirchen an, was bieten die ganzen sozialen Einrichtungen in der Nähe an, dass man einfach sagen kann: ‚Ah, Sie interessiert das, das Angebot gibt's da.' Das ist mir tatsächlich sehr wichtig, [dass ich darüber informiere], unabhängig von mir als Einrichtung." (Int2-ASZ-F, Pos. 69)

Vom Gespräch zur Fallzahl: Statistik und Dokumentation

Die Tätigkeiten der SAVE-Fachkräfte umfassen auch die Dokumentation der Begegnungen im Sozialraum und die anschließende Übertragung der so entstandenen Kontakte in die SAVE-Statistik. Die SAVE-Fachkräfte haben im Rahmen ihrer täglichen Praxis individuelle und kreative Strategien zur Dokumentation ihrer Kontakte entwickelt.

Beispielzitat

„Das hab ich ziemlich schnell und von Anfang an so gemacht, dass ich die Notizbuchfunktion in meinem Smartphone genutzt habe und immer, wenn die Leute dann weg waren oder wenn ich weitergegangen bin, mir da draufgesprochen habe. Das hat sich sehr bewährt. Da schreib ich z.B. „1. Mai" und dann „ältere Dame, Rollator, hatte Frage zur Pflegeversicherung, war aufgeschlossen, war gehbehindert", also, das sind so meine sehr subjektiven Bemerkungen. Aber so, dass ich mich [nachher] noch dran erinnern kann, wer das war." (Int3-ASZ-F, Pos. 129)

Für die Straße: Standortbestimmung und Zukunftsperspektiven

**Die Phase der Einarbeitung: Zum gelingenden Start in die SAVE-Arbeit**
Zum Start des SAVE-Projekts sahen sich die Fachkräfte mit der Aufgabe konfrontiert, eine neue berufliche Rolle auszufüllen, ohne dabei auf bestehende Erfahrungen zurückgreifen zu können. Dies bedeutete für viele von ihnen, dass sie zunächst einmal ihren eigenen Arbeitsstil und -rhythmus entwickeln mussten. Einerseits bot diese Pionierarbeit die Möglichkeit, individuelle innovative Ansätze zu verfolgen. Andererseits fehlten etablierte Strukturen und bewährte Methoden, was teilweise den Einstieg in die neue Tätigkeit erschwerte und Unsicherheiten mit sich brachte.
**Beispielzitat**
„Ich hab [mir am Anfang] gesagt, gut, ich lerne jetzt erstmal die ASZ-Arbeit kennen. Dann bin ich die ersten Male auf die Straße gegangen, und dabei macht man sich ja auch [...] viele Gedanken [...] und kann auch so ein bisschen Ideen entwickeln. Und dann muss man aber eigentlich zu dem Punkt kommen, dass man sagt, jetzt brauchen wir aber ein Konzept mit den Kollegen, dass man auch gemeinsam was entwickelt und [SAVE] dann auch was Gemeinsames wird. Schwierig ist es, wenn SAVE dann [die Aufgabe von] einem alleine bleibt." (Int3-ASZ-F, Pos. 41)

**Zusammen ist man weniger allein: Tandems, Teams und Patenschaften**
Eine wertschätzende und kooperative Zusammenarbeit mit dem ASZ-Team und der ASZ-Leitung ist von wesentlicher Bedeutung für den langfristigen Erfolg von SAVE. Die SAVE-Fachkräfte profitieren von verschiedenen Formen der Zusammenarbeit. In einem ASZ teilen sich zwei Fachkräfte ihre Stelle und sind also sogenanntes „SAVE-Tandem" in ihrem Stadtteil unterwegs, was von allen Beteiligten als sehr hilfreich empfunden wird. Externe Unterstützung erhalten die SAVE-Fachkräfte außerdem durch den Austausch mit den anderen SAVE-Fachkräften und ein spezifisches Patensystem.
**Beispielzitat**
„Meine [SAVE-]Kolleginnen [in anderen Stadtvierteln] konnten mir [...] ganz viel weiterhelfen bei allem, was Statistik oder so anbelangt. Oder generell auch [in Bezug darauf], wie angesprochen wird. Ich hab natürlich dann auch ein bisschen meine eigene Art und Weise reingebracht. Aber einfach zu sehen, wie da auf Leute zugegangen wird, wie ein Gespräch verlaufen kann, das war natürlich wahnsinnig wertvoll. Und auch [einfach die Tatsache,] jemanden als Ansprechpartner zu haben." (Int1-ASZ-F, Pos. 63)

(Keine) Ressourcen für die SAVE-Klientel?

Eine häufig diskutierte Frage betrifft das Thema der (nicht) vorhandenen Ressourcen für die Weitervermittlung der SAVE-Klientel. Neben der Überlastung externer Hilfsangebote stehen die SAVE-Fachkräfte zusätzlich vor dem Problem begrenzter interner Kapazitäten. Viele ASZ-Kurse und -Angebote sind voll, der soziale Mittagstisch oft ausgebucht und die Nachfrage nach Beratungsterminen ist hoch.

Beispielzitat

„Das ist so eine Gratwanderung, [...], die Seniorinnen und Senioren mit diesen Multiproblemen, holen wir uns die hier rein ins Alten- und Service-Zentrum, sind wir da noch die richtige Einrichtung? Und was machen wir, wenn uns aber diese Notlage bekannt ist? Da kann man ja dann auch nicht sagen, da können wir jetzt nix machen. Also, wo geben wir die dann auch hin ab, welche Einrichtungen sind dann da die richtigen? Und auch den Kontakt dahin zu schaffen, das ist auch nicht so einfach [...].“ (Int3-ASZ-L, Pos. 312)

Verschiedene Viertel, verschiedene Bedarfe!

Der nachhaltige Erfolg von SAVE hängt maßgeblich von der Anpassung des Angebots an die spezifischen Bedürfnisse der einzelnen Stadtteile ab. Da die Münchner Stadtbezirke sozialräumlich sehr unterschiedlich strukturiert sind und sich flächenmäßig stark unterscheiden, müssen diese Unterschiede bei der Implementierung von SAVE berücksichtigt werden. Besonders große Stadtbezirke benötigen individuelle Anpassungsstrategien. Einige SAVE-Fachkräfte konzentrieren sich auf bestimmt Straßenzüge und Knotenpunkte und mussten mehrere unterschiedliche Routen entwickeln. Andere Fachkräfte nutzen die Angebote von Netzwerkpartner*innen, um in weiter entfernten Gebieten anzudocken und mit älteren Menschen in Kontakt zu treten.

Beispielzitat

„Eine Sache hat sich jetzt [schon] fest installiert. Und zwar hat dieses ASZ hier auch nochmal ein Essensangebot in X. Und da gehe ich jetzt immer mittwochs mittags hin und esse da mit. [...] Und auch um dieses Essensangebot rum, das findet in einer Kirche statt, und da drum rum ist auch so ein kleines Zentrum mit Geschäften, Friseur, Sparkasse usw. Also, so ein kleiner Mittelpunkt von den Menschen, die dort leben. Und dazu gehören viele Senioren. Also, ich treffe da sehr viele Seniorinnen an, und auch da ergeben sich Sachen [...]. Das ist alles noch in den Kinderschuhen, aber ich merke, es wächst was.“ (Int4-ASZ-F, Pos. 15)
